# Powder X-ray investigation of 4,4′-diisocyano-3,3′-dimethyl­biphen­yl

**DOI:** 10.1107/S1600536813004315

**Published:** 2013-02-20

**Authors:** Mwaffak Rukiah, Mahmoud Al-Ktaifani

**Affiliations:** a Department of Chemistry, Atomic Energy Commission of Syria (AECS), PO Box 6091, Damascus, Syrian Arab Republic

## Abstract

The title compound, C_16_H_12_N_2_, was investigated in a powder diffraction study and its structure refined utilizing the Rietveld Method. The mol­ecule has approximate *C*2 symmetry. The dihedral angle between the rings is 25.6 (7)°. The crystal packing is consolidated by weak C—H⋯C N hydrogen-bond-like contacts, which lead to the formation of a three-dimensional network. Further stabilization of the crystal structure is achived by weak non-covalent π–π inter­actions between aromatic rings, with a centroid–centroid distance of 3.839 (8) Å.

## Related literature

For disocyano ligands and their coordination complexes, see: Harvey (2001 ▶); Sakata *et al.* (2003 ▶); Espinet *et al.* (2000 ▶); Moigno *et al.* (2002 ▶). For the preparation of the bidentate ligand CNCH_2_C(CH_3_)_2_CH_2_NC and its organometallic polymeric structures, see: Al-Ktaifani *et al.* (2008 ▶); Rukiah & Al-Ktaifani (2008 ▶, 2009 ▶); Al-Ktaifani & Rukiah (2010 ▶). For chelate complexing, see: Chemin *et al.* (1996 ▶). For the structure of isocyanide, see: Lentz & Preugschat (1993 ▶). For practical applications of oganometallic complexes with diisocyanide ligands, see: Fortin *et al.* (2000 ▶). For standard bond-lengths, see: Allen *et al.* (1987 ▶). For background and details of methods applied in powder diffraction, see: Boultif & Louër (2004 ▶); Rodriguez-Carvajal (2001 ▶); Roisnel & Rodriguez-Carvajal (2001 ▶); Le Bail *et al.* (1988 ▶); Toby (2001 ▶); Thompson *et al.* (1987 ▶); Finger *et al.* (1994 ▶); Stephens (1999 ▶). 
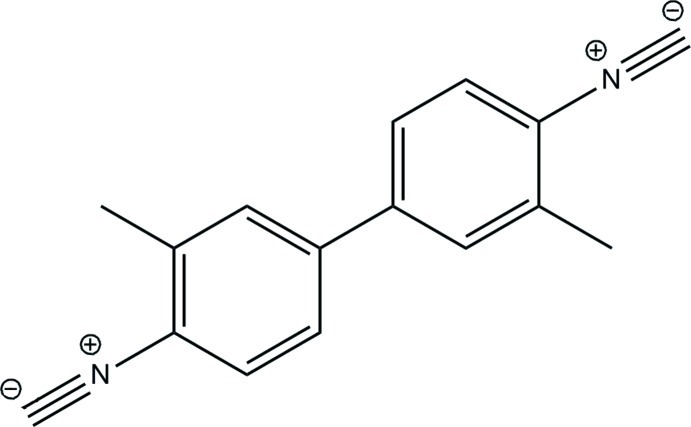


## Experimental

### 

#### Crystal data

C_16_H_12_N_2_
*M* *_r_* = 232.28
Monoclinic, 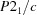

*a* = 11.9045 (4) Å
*b* = 14.6235 (4) Å
*c* = 7.61672 (15) Å
β = 105.483 (2)°
*V* = 1277.84 (7) Å^3^
*Z* = 4
Cu *K*α_1_ radiation
λ = 1.5406 Å
μ = 0.56 mm^−1^
*T* = 298 K
flat sheet, 8 × 8 mm

#### Data collection

STOE Transmission STADI P diffractometer
Specimen mounting: powder loaded between two Mylar foils
Data collection mode: transmission
Scan method: step
Absorption correction: for a cylinder mounted on the ϕ axis (*GSAS*; Larson & Von Dreele, 2004 ▶)*T*_min_ = 0.685, *T*_max_ = 0.767
2θ_min_ = 4.999°, 2θ_max_ = 89.979°, 2θ_step_ = 0.02°

#### Refinement

*R*_p_ = 0.016
*R*_wp_ = 0.021
*R*_exp_ = 0.016
*R*(*F* ^2^) = 0.026
χ^2^ = 1.742
4250 data points
120 parameters
H-atom parameters not refined

### 

Data collection: *WinXPOW* (Stoe & Cie, 1999 ▶); cell refinement: *GSAS* (Larson & Von Dreele, 2004 ▶); data reduction: *WinXPOW*; program(s) used to solve structure: *FOX* (Favre-Nicolin & Černý, 2002 ▶); program(s) used to refine structure: *GSAS*; molecular graphics: *ORTEP-3* (Farrugia, 2012 ▶); software used to prepare material for publication: *publCIF* (Westrip, 2010 ▶).

## Supplementary Material

Crystal structure: contains datablock(s) global, I. DOI: 10.1107/S1600536813004315/lh5582sup1.cif

Rietveld powder data: contains datablock(s) I. DOI: 10.1107/S1600536813004315/lh5582Isup2.rtv

Supplementary material file. DOI: 10.1107/S1600536813004315/lh5582Isup3.cml

Additional supplementary materials:  crystallographic information; 3D view; checkCIF report

## Figures and Tables

**Table 1:** Hydrogen-bond geometry (Å, °)

*D*—H⋯*A*	*D*—H	H⋯*A*	*D*⋯*A*	*D*—H⋯*A*
C6—H6⋯C14^i^	0.991	2.900	3.69 (2)	137.36
C7—H7⋯C14^ii^	0.986	2.815	3.737 (17)	155.86
C16—H16*b*⋯C1^iii^	0.989	2.809	3.73 (2)	154.52

Symmetry codes: (i) 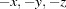; (ii) 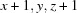; (iii) 
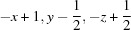
.

**Fig. 1.**
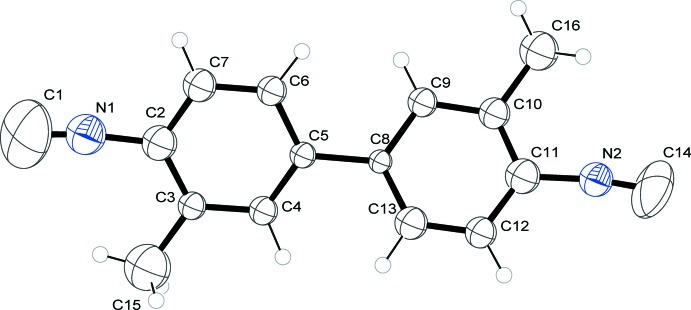
The asymmetric unit of (I) with displacement ellipsoids drawn at the 50% probability level. H atoms are represented as small spheres of arbitrary radii.

**Fig. 2.**
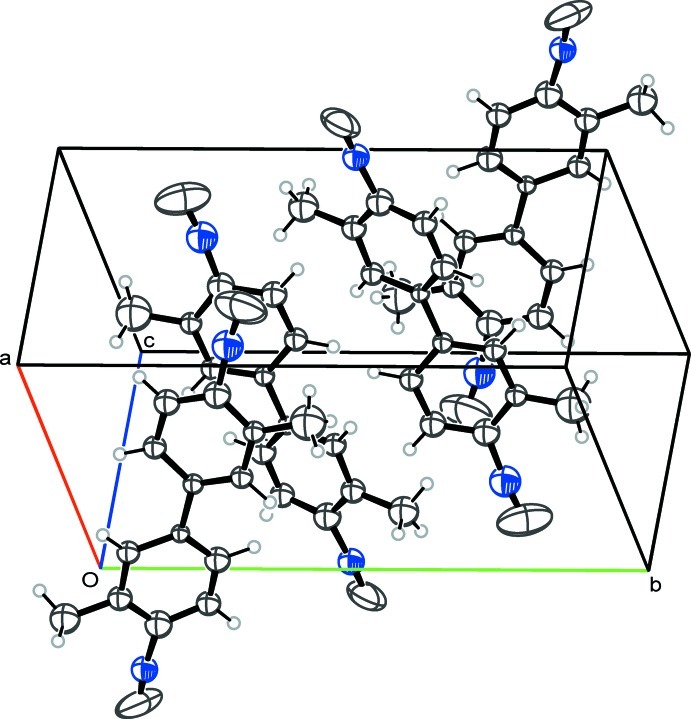
View of the crystal packing of compound (I).

**Fig. 3.**
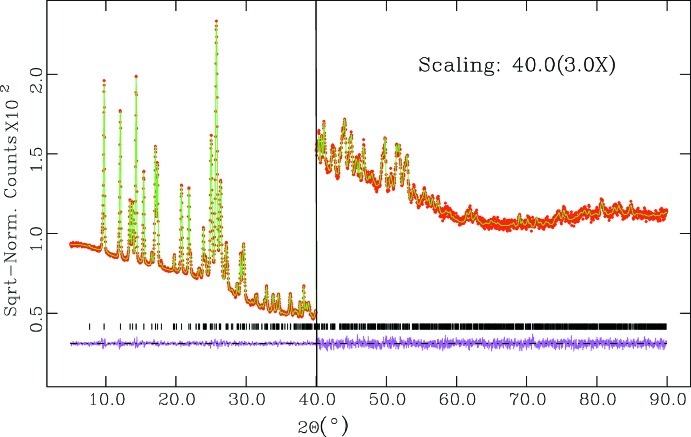
Final Rietveld plot of compound (I). Observed data points are indicated by dots, the best-fit profile (upper trace) and the difference pattern (lower trace) are solid lines. The vertical bars indicate the positions of Bragg peaks.

## supplementary crystallographic information

### Comment

Over the past decade a new rich area of organometallic chemistry has been developed in which diisocyanides have been used as potential bridging ligands in the synthesis of bi- and tri- and tetra nuclear complexes and organometallic polymers (Harvey, 2001; Sakata *et al.*, 2003; Espinet *et al.*, 2000; Moigno *et al.*, 2002). These new materials have been reported to have practical potential applications in different fields, such as semi- and photoconductivity and photovoltaic cells (Fortin *et al.*, 2000). Very recently, in a series of publications we have utilized the bidentate ligand 2,2-dimethylpropane-1,3-diyl diisocyanide in the syntheses of the organometallic polymers [Ag(C_7_H_10_N_2_)(*X*)]_n_ (*X* = Cl^-^, Br^-^, I^-^ or NO_3_^-^) (Al-Ktaifani *et al.*, 2008; Al-Ktaifani & Rukiah, 2010; Rukiah & Al-Ktaifani, 2008; Rukiah &Al-Ktaifani, 2009), which have been completely characterized by X-ray powder diffraction studies, IR spectroscopy and micro-analysis. A major point of interest in the previously reported polymers {Ag(I)[CNCH_2_C(CH_3_)_2_CH_2_NC]*X*}_n_ (*X* = Cl^-^, I^-^ , Br^-^ or NO_3_^-^) is their similar polymeric structures regardless of the counterpart anions. Also noteworthy is the bidentate ligand exhibits a very strong tendency to form polymeric complexes rather than dimeric or trimeric complexes suggesting the 2,2-dimethylpropane-1,3-diyl diisocyanide to be a potential bidentate bridging ligand in the syntheses of organometallic polymers of different transition metals. In similar manner, the bidentate bridging ligand 4,4`-diisocyano-3,3`-dimethyl-biphenyl was prepared in order to be utilized in the synthesis of new organometallic complexes. The syntheses of new organometallic complexes of this bidentate bridging ligand and their solid state characterizations are currently under investigations.

In this article, the solid state characterization of the bidentate bridging ligand 4,4`-diisocyano-3,3`-dimethyl-biphenyl (I) is presented. In contrast to the extensively structurally characterized diisocyanide complexes, reports of the molecular structures of free diisocyanides are rare. This was an incentive to described the molecular structure of the uncomplexed diisocyanide 4,4'-diisocyano-3,3'-dimethylbiphenyl, C_16_H_12_N_2_, by powder X-ray diffraction study.

Compound (I) has a tendency to crystalize in the form of very fine beige powder. Since no single-crystal of sufficient thickness and quality could be obtained, a structure determination by powder X-ray diffraction data was attempted. A view of the molecular structure is shown in Fig. 1. Compound (I) crystallizes with one molecule in the asymmetric unit, having a approximate C_2_ symmetry. In the crystal structure of (I), weak non classical intermolecular hydrogen-bond-like contacts C—H···C (Table 1) between the carbon centre of CH_3_ or aromatic ring and the carbon center of cyanide group were observed . These contacts link the molecules to form a three-dimensional network and may be effective in the stabilization of the crystal structure (Fig. 2). The crystal packing of (I) is also further stabilized by noncovalent weak π—π aromatic interactions between phenyl rings of adjacent molecule [minimum centroid—centroid distances between two adjacent (but crystallographically different) *Cg*1···*Cg*1(*x*, 1/2 - *y*, -1/2 - *z*) = 3.839 (8) Å and *Cg*1···*Cg*1(*x*, 1/2 - *y*, 1/2 + *z*) = 3.838 (8) Å, where *Cg*1 is the centroid of the phenyl C2—C7 ring]. It is notworthy that a contact between two adjecent methyl groups is most likely repulsive and possibly even destabilizing C16···C16(-x, -y, -z-1) = 3.68 (2)Å. All bond distances (Allen *et al.*, 1987) and angles in compound (I) are in their normal ranges.

### Experimental

All reactions and manipulations were carried out in air with reagent grade solvent. 4,4'-diamino-3,3'-dimethylbiphenyl (*o*-tolidine) was a commercial sample and was used as received. IR spectra were operated on FTIR Thermo Nicolet 6700. Powder X-ray diffraction was performed by Stoe Transmission diffractometre (Stadi P). A round bottom flask was charged with *o*-tolidine (10 g, 47 mmol), KOH (50%, 50 ml), CH_2_Cl_2_ (75 ml) and benzyltriethylammonium chloride (5.3 mmol, 1.2 g). To the mixture was added dropwise CHCl_3_ (10 ml). The resultant mixture was left to reflux spontaneously and stirred over night. The solution was filtered and diluted with 200 ml of H_2_O and the product extracted with CH_2_Cl_2_. The organic layer was separated, washed with 100 ml of H_2_O. The organic layer was dried over anhydrous Na_2_SO_4_, solvent removed, washed with Et_2_O to give beige powder. The product was purified by re-crystallization from CH_2_Cl_2_ to give light brown powder (3 g, yield 25%, m.p. 389 K). Spectroscopic analysis: IR (KBr, *ν*, cm^-1^): 2124.4 (N≡C); ^1^H NMR (400 MHz, CDCl_3_, 298 K) *δ* 7.39–7.47 (*m*, aromatic, 6H), 2.52 (*s*, CH_3_, 6H).^13^C{1H} NMR (100 MHz, CDCl_3_, 298 K) *δ* 166.91 (*s*, N≡ C), 140.55 (*s*, aromatic), 135.59 (*s*, aromatic), 129.19 (*s*, aromatic), 127.05 (*s*, aromatic), 126.15 (*s*, aromatic), 125.43 (*s*, aromatic), 18.78 (*s*, CH_3_).

### Refinement

No geometric soft restraints were applied during the Rietveld refinement. The methyl and aromatic H atoms were positioned in their idealized geometries. The coordinates of these H atoms were not refined. We used constant isotropic displacement parameters (0.05 Å^2^) for the aromatic H atoms and (0.1 Å^2^) for methyl H atoms.The final refinement cycles were performed using anisotropic atomic displacement parameters for the carbon of cyano group. The final Rietveld plot of the X-ray diffraction pattern is given in Fig. 3.

### Crystal data

**Table:**

C_16_H_12_N_2_	*F*(000) = 488
*M**_r_* = 232.28	*D*_x_ = 1.207 Mg m^−^^3^
Monoclinic, *P*2_1_/*c*	Cu *K*α_1_ radiation, λ = 1.5406 Å
Hall symbol: -P 2ybc	µ = 0.56 mm^−^^1^
*a* = 11.9045 (4) Å	*T* = 298 K
*b* = 14.6235 (4) Å	Particle morphology: Fine powder
*c* = 7.61672 (15) Å	light_brown
β = 105.483 (2)°	flat sheet, 8 × 8 mm
*V* = 1277.84 (7) Å^3^	Specimen preparation: Prepared at 298 K and 101.3 kPa
*Z* = 4	

### Data collection

**Table:**

STOE Transmission STADI P diffractometer	Scan method: step
Radiation source: sealed X-ray tube	Absorption correction: for a cylinder mounted on the φ axis *GSAS* Absorption/surface roughness correction: function number 4 Flat plate transmission absorption correction Terms = 0.17220 0.0000 Correction is not refined.
Curved Ge(111) monochromator	*T*_min_ = 0.685, *T*_max_ = 0.767
Specimen mounting: powder loaded between two Mylar foils	2θ_min_ = 4.999°, 2θ_max_ = 89.979°, 2θ_step_ = 0.02°
Data collection mode: transmission	

### Refinement

**Table:**

Least-squares matrix: full	Excluded region(s): none
*R*_p_ = 0.016	Profile function: CW Profile function number 4 with 21 terms Pseudovoigt profile coefficients as parameterized in (Thompson *et al.*,1987.Asymmetry correction of Finger *et al.*(Finger *et al.*,1994). Microstrain broadening by Stephens(Stephens, 1999). #1(GU) = 0.000 #2(GV) = 0.000 #3(GW) = 10.785 #4(GP) = 0.000 #5(LX) = 2.472 #6(ptec) = 0.00 #7(trns) = 0.00 #8(shft) = 0.0000 #9(sfec) = 0.00 #10(S/L) = 0.0225 #11(H/L) = 0.0225 #12(eta) = 0.4987 #13(S400 ) = 9.0E-02 #14(S040 ) = 1.1E-02 #15(S004 ) = 2.8E+00 #16(S220 ) = 1.9E-02 #17(S202 ) = -1.5E-02 #18(S022 ) = 3.5E-02 #19(S301 ) = -2.0E-01 #20(S103 ) = 1.1E+00 #21(S121 ) = -6.1E-02 Peak tails are ignored where the intensity is below 0.0010 times the peak Aniso. broadening axis 0.0 0.0 1.0
*R*_wp_ = 0.021	120 parameters
*R*_exp_ = 0.016	0 restraints
*R*(*F*^2^) = 0.02609	H-atom parameters not refined
χ^2^ = 1.742	(Δ/σ)_max_ = 0.02
4250 data points	Background function: GSAS Background function number 1 with 20 terms. Shifted Chebyshev function of 1st kind 1: 3509.16 2: -3547.18 3: 1729.31 4: -271.390 5: -158.493 6: 133.640 7: -41.5743 8: -87.6309 9: 73.0201 10: 78.1201 11: -126.319 12: 73.5124 13: 5.65073 14: -38.9623 15: 13.5948 16: 6.87496 17: -4.39687 18: 0.392461 19: 8.77696 20: -6.87661

### Special details

**Table:**

Experimental. The sample was ground lightly in a mortar, loaded between two Mylar foils and fixed in the sample holder with a mask of 8.0 mm internal diameter.
Refinement. For pattern indexing, the extraction of the peak positions was carried out with the program*WinPLOTR* (Roisnel & Rodriguez-Carvajal, 2001). Pattern indexing was performed with the program *DicVol4.0* (Boultif & Louër, 2004). The first 20 lines of powder pattern were completely indexed on the basis of monoclinic system. The absolute error on each observed line was fixed at 0.02° (2θ). The figures of merit are sufficiently high to support the obtained indexing results [*M*(20) = 22.5, *F*(20) = 41.1(0.0087, 56)]. The whole powder diffraction pattern from 5 to 90° (2θ) was subsequently refined with cell and resolution constraints (Le Bail *et al.*, 1988) with a space group without systematic extinctions in monoclinic system, *P2/m*, using the "profile matching" option of the program *FullProf* (Rodriguez-Carvajal, 2001). The best estimated space group in the monoclinic system was *P2_1_/c* which determined with the help of the program *Check Group* interfaced by *WinPLOTR* (Roisnel & Rodriguez, 2001). The number of molecules per unit cell was estimated to be equal to *Z* = 4, it can be concluded that the number of molecules in the asymmetric unit is *Z' =* 1 for the space group *P2_1_/c*.The structure was solved *ab initio* by direct space method (Monte Carlo simulated annealing with parallel tempering algorithm) using the program *FOX* (Favre-Nicolin & Černý, 2002). The model found by this program was introduced in the program *GSAS* (Larson & Von Dreele, 2004) implemented in *EXPGUI* (Toby, 2001) for Rietveld refinements. The background was refined using a shifted Chebyshev polynomial with 20 coefficients. During the Rietveld refinements, the effect of the asymmetry of peaks was corrected using a pseudo-Voigt description of the peak shape (Thompson *et al.*, 1987) which allows for angle-dependent asymmetry with axial divergence (Finger *et al.*, 1994). The two asymmetry parameters of this function *S/L* and *D/L* were both fixed at 0.0225 during the Rietveld refinement. Intensities were corrected from absorption effects with a µ*.d* value of 0.1722.

### Fractional atomic coordinates and isotropic or equivalent isotropic displacement parameters (Å^2^)

**Table:**

	*x*	*y*	*z*	*U*_iso_*/*U*_eq_	
C1	0.7040 (15)	0.3220 (13)	0.546 (3)	0.13957	
C2	0.5080 (12)	0.2651 (12)	0.326 (2)	0.049 (7)*	
C3	0.4211 (14)	0.3265 (8)	0.237 (2)	0.031 (5)*	
C4	0.3141 (13)	0.2954 (10)	0.1285 (17)	0.033 (5)*	
C5	0.2881 (12)	0.2038 (10)	0.120 (2)	0.027 (5)*	
C6	0.3738 (13)	0.1398 (8)	0.209 (2)	0.035 (6)*	
C7	0.4835 (12)	0.1710 (10)	0.3129 (17)	0.044 (6)*	
C8	0.1712 (13)	0.1714 (10)	0.0015 (19)	0.022 (5)*	
C9	0.1588 (12)	0.0804 (10)	−0.075 (2)	0.034 (5)*	
C10	0.0455 (16)	0.0559 (9)	−0.181 (2)	0.039 (6)*	
C11	−0.0471 (12)	0.1147 (11)	−0.213 (2)	0.049 (6)*	
C12	−0.0348 (12)	0.2013 (10)	−0.1276 (19)	0.039 (6)*	
C13	0.0750 (14)	0.2270 (7)	−0.030 (2)	0.043 (6)*	
C14	−0.2511 (14)	0.0608 (10)	−0.386 (2)	0.11783	
C15	0.4492 (9)	0.4276 (9)	0.2552 (18)	0.086 (6)*	
C16	0.0322 (13)	−0.0402 (9)	−0.2630 (19)	0.060 (5)*	
N1	0.6187 (12)	0.2921 (10)	0.449 (2)	0.065 (7)*	
N2	−0.1575 (11)	0.0800 (9)	−0.3173 (19)	0.046 (6)*	
H4	0.25719	0.3431	0.06671	0.05*	
H6	0.35313	0.07403	0.19577	0.05*	
H7	0.53998	0.12458	0.37635	0.05*	
H9	0.2264	0.04273	−0.05912	0.05*	
H12	−0.10372	0.23895	−0.1533	0.05*	
H13	0.08075	0.29047	0.02542	0.05*	
H15a	0.438	0.45236	0.13397	0.1*	
H15b	0.39451	0.45559	0.3131	0.1*	
H15c	0.52758	0.43661	0.32669	0.1*	
H16a	0.04874	−0.04178	−0.38138	0.1*	
H16b	0.08445	−0.08471	−0.18326	0.1*	
H16c	−0.04778	−0.06116	−0.2777	0.1*	

### Atomic displacement parameters (Å^2^)

**Table:**

	*U*^11^	*U*^22^	*U*^33^	*U*^12^	*U*^13^	*U*^23^
C1	0.13 (2)	0.21 (2)	0.07 (2)	0.001 (18)	0.004 (15)	−0.048 (17)
C14	0.081 (18)	0.127 (18)	0.11 (2)	−0.014 (14)	−0.033 (19)	0.063 (15)

### Geometric parameters (Å, º)

**Table:**

C1—N1	1.171 (19)	C5—C8	1.518 (13)
N1—C2	1.451 (17)	C8—C9	1.446 (14)
C2—C3	1.402 (18)	C9—C10	1.421 (18)
C3—C4	1.397 (17)	C9—H9	0.956
C3—C15	1.513 (14)	C10—C11	1.367 (16)
C15—H15a	0.967	C10—C16	1.528 (19)
C15—H15b	0.969	C16—H16a	0.973
C15—H15c	0.956	C16—H16b	0.989
C4—C5	1.372 (13)	C16—H16c	0.978
C4—H4	0.998	C11—C12	1.414 (14)
C5—C6	1.416 (14)	C11—N2	1.435 (15)
C6—C7	1.410 (14)	C12—C13	1.373 (14)
C6—H6	0.992	C12—H12	0.964
C7—C2	1.404 (14)	C13—H13	1.014
C7—H7	0.987	C14—N2	1.133 (15)
			
C3—C2—C7	118.7 (14)	C9—C10—C16	116.3 (18)
C3—C2—N1	124.3 (17)	C11—C10—C16	121.0 (18)
C7—C2—N1	116.7 (19)	C10—C11—C12	120.1 (15)
C2—C3—C4	121.1 (12)	C10—C11—N2	116.9 (18)
C2—C3—C15	117.7 (16)	C12—C11—N2	122.6 (18)
C4—C3—C15	121.2 (16)	C11—C12—C13	117.5 (14)
C3—C4—C5	120.3 (14)	C11—C12—H12	116.1
C3—C4—H4	116.6	C13—C12—H12	126.1
C5—C4—H4	123.0	C8—C13—C12	124.2 (15)
C4—C5—C6	119.9 (15)	C8—C13—H13	120.6
C4—C5—C8	119.5 (16)	C12—C13—H13	115.1
C6—C5—C8	120.5 (13)	C3—C15—H15a	107.9
C5—C6—C7	119.7 (14)	C3—C15—H15b	107.2
C5—C6—H6	117.7	C3—C15—H15c	110.2
C7—C6—H6	122.5	H15a—C15—H15b	109.7
C2—C7—C6	120.1 (13)	H15a—C15—H15c	110.9
C2—C7—H7	122.5	H15b—C15—H15c	110.8
C6—C7—H7	117.4	C10—C16—H16a	112.1
C5—C8—C9	120.4 (13)	C10—C16—H16b	112.0
C5—C8—C13	120.6 (15)	C10—C16—H16c	109.1
C9—C8—C13	119.0 (16)	H16a—C16—H16b	107.6
C8—C9—C10	116.1 (14)	H16a—C16—H16c	108.6
C8—C9—H9	119.2	H16b—C16—H16c	107.3
C10—C9—H9	124.6	C1—N1—C2	174 (3)
C9—C10—C11	122.7 (14)	C11—N2—C14	171 (2)
			
N1—C2—C3—C4	−176.4 (14)	C6—C5—C8—C13	156.0 (15)
N1—C2—C3—C15	6 (2)	C5—C6—C7—C2	−1 (2)
C7—C2—C3—C4	−4 (2)	C5—C8—C9—C10	178.8 (13)
C7—C2—C3—C15	179.3 (13)	C13—C8—C9—C10	1 (2)
N1—C2—C7—C6	174.5 (13)	C5—C8—C13—C12	−176.6 (14)
C3—C2—C7—C6	1 (2)	C9—C8—C13—C12	2 (2)
C2—C3—C4—C5	6 (2)	C8—C9—C10—C11	1 (2)
C15—C3—C4—C5	−177.1 (13)	C8—C9—C10—C16	179.7 (13)
C3—C4—C5—C6	−5 (2)	C9—C10—C11—N2	−178.0 (14)
C3—C4—C5—C8	179.9 (13)	C9—C10—C11—C12	−5 (2)
C4—C5—C6—C7	3 (2)	C16—C10—C11—N2	4 (2)
C8—C5—C6—C7	177.6 (13)	C16—C10—C11—C12	176.4 (13)
C4—C5—C8—C9	152.6 (14)	N2—C11—C12—C13	179.5 (14)
C4—C5—C8—C13	−29 (2)	C10—C11—C12—C13	7 (2)
C6—C5—C8—C9	−22 (2)	C11—C12—C13—C8	−5 (2)

### Hydrogen-bond geometry (Å, º)

**Table:**

*D*—H···*A*	*D*—H	H···*A*	*D*···*A*	*D*—H···*A*
C6—H6···C14^i^	0.991	2.900	3.69 (2)	137.36
C7—H7···C14^ii^	0.986	2.815	3.737 (17)	155.86
C16—H16*b*···C1^iii^	0.989	2.809	3.73 (2)	154.52
C16—H16*a*···C16^iv^	0.973	2.883	3.68 (2)	139.57

Symmetry codes: (i) −*x*, −*y*, −*z*; (ii) *x*+1, *y*, *z*+1; (iii) −*x*+1, *y*−1/2, −*z*+1/2; (iv) −*x*, −*y*, −*z*−1.
